# Family-centred interventions by primary healthcare services for Indigenous early childhood wellbeing in Australia, Canada, New Zealand and the United States: a systematic scoping review

**DOI:** 10.1186/s12884-017-1247-2

**Published:** 2017-02-21

**Authors:** Janya McCalman, Marion Heyeres, Sandra Campbell, Roxanne Bainbridge, Catherine Chamberlain, Natalie Strobel, Alan Ruben

**Affiliations:** 1 Central Queensland University, Cairns, Australia; 2 James Cook University, Cairns, Australia; 3 University of Melbourne, Melbourne, Australia; 4 University of Western Australia, Crawley, Australia; 5 Apunipima Cape York Health Council, Bungalow, Australia

**Keywords:** Family-centred, Patient-centred, Indigenous, Maternal and child health, Health outcomes

## Abstract

**Background:**

Primary healthcare services in Australia, Canada, New Zealand and the United States have embraced the concept of family-centred care as a promising approach to supporting and caring for the health of young Indigenous children and their families. This scoping review assesses the quality of the evidence base and identifies the published literature on family- centred interventions for Indigenous early childhood wellbeing.

**Methods:**

Fourteen electronic databases, grey literature sources and the reference lists of Indigenous maternal and child health reviews were searched to identify relevant publications from 2000 to 2015. Studies were included if the intervention was: 1) focussed on Indigenous children aged from conception to 5 years from the abovementioned countries; 2) led by a primary healthcare service; 3) described or evaluated; and 4) scored greater than 50% against a validated scale for family-centredness. The study characteristics were extracted and quality rated. Reported aims, strategies, enablers and outcomes of family-centredcare were identified using grounded theory methods.

**Results:**

Eighteen studies (reported in 25 publications) were included. Three were randomised controlled studies; most were qualitative and exploratory in design. More than half of the publications were published from 2012 to 2015. The overarching aim of interventions was to promote healthy families. Six key strategies were to: support family behaviours and self- care, increase maternal knowledge, strengthen links with the clinic, build the Indigenous workforce, promote cultural/ community connectedness and advocate for social determinants of health. Four enablers were: competent and compassionate program deliverers, flexibility of access, continuity and integration of healthcare, and culturally supportive care. Health outcomes were reported for Indigenous children (nutritional status; emotional/behavioural; and prevention of injury and illness); parents/caregivers (depression and substance abuse; and parenting knowledge, confidence and skills); health services (satisfaction; access, utilization and cost) and community/cultural revitalisation.

**Discussion and conclusion:**

The evidence for family-centred interventions is in the early stages of development, but suggests promise for generating diverse healthcare outcomes for Indigenous children and their parents/caregivers, as well as satisfaction with and utilisation of healthcare, and community/cultural revitalisation. Further research pertaining to the role of fathers in family-centred care, and the effects and costs of interventions is needed.

## Background

Primary healthcare services have embraced the concept of family-centred models of care as one approach to improve health and preventive services for Indigenous children [1–3]. Family-centred approaches differ from traditional maternal and child healthcare which focus on the management of individual women’s pregnancies and infants’ health and development at healthcare clinics. Instead, family-centred care approaches provide support and care for the whole family, their lives and wellbeing concerns, often at the family’s home.

This scoping review was conducted to inform the development for a Cochrane review protocol [3] by systematically searching, selecting and synthesizing existing knowledge to map key concepts, types of evidence, and gaps in research about family-centred healthcare [4]. As suggested by Dijkers [5], assessments of the quality of the primary studies are included to provide confidence that the implications of the review for policy, practice or patients are based on high quality research. The research question was: What is thecurrent evidence base for the impact of family-centred interventions on Indigenous earlychildhood health? Both the Cochrane and this scoping review were contracted by a Queensland regional Indigenous community controlled health service, Apunipima Cape York Health Council, to inform the implementation of their family-centred Baby One Program (Bainbridge R, McCalman J, Campbell C, Redman-MacLaren M, Vine K, Canuto K, Sewter J, MacDonald M: Growing a relational and responsive family health promotion program: A grounded theory evaluation of the Baby One Program, inpreparation).

In mainstream populations, many health care providers now recognise family-centred care and the related concept of patient-centred care as integral to patient health, satisfaction, and health care quality, and consider them to be the standard of child health care [6]. For example, the US Healthy People 2020 plan for children recommends that children with special health care needs should receive care in a “family-centred, comprehensive, coordinated system” [7]. There is evidence from mainstream settings that family-centred interventions have resulted in decreased depression rates and burden in carers, improved quality of life for the entire family and satisfaction with care, as well as greater health service effectiveness and efficiency with reduced cost [8].

The need for improved child healthcare for Indigenous populations is evidenced by persistent disparities in child health equity in Australia, Canada, New Zealand and the United States. Mortality rates are higher in the four countries for all Indigenous infants except Native Hawaiians; there are generally fewer children born with normal birthweights (between 2500 and 4500 g); and childhood obesity rates are considerably higher for Indigenous than the general populations in each of these countries [1]. These disparities reflect the shared legacy of the impacts of colonisation in these countries; whereby exclusionary social policies have to varying degrees disrupted family relations, continuity and functioning [9].

Many Indigenous families deal with ongoing stressors, which can manifest inpsychological distress, grief, smoking and alcohol and drug misuse, mental illnesses, and/or violence; and thus their ability to nurture children [9]. In turn, families can experience issues such as lack of food security, child neglect, and the removal of children [10]. However, Indigenous families also commonly experience strengths, such as strong bonding capital associated with their inclusion of members of their extended families, and the influence of traditional cultural norms on child rearing practices [9]. These strengths provide opportunity upon which engagement in health promoting family-centred approaches with services can be built to support improvements both to family lifestyle factors but also on the upstream social determinants of Indigenous childrens’ health and wellbeing [9].

Primary health care services in Indigenous communities, which are increasingly managed and delivered by Indigenous community controlled health services, have taken opportunities to develop and implement family-centred interventions to improve Indigenous child health. By ensuring that care is planned and implemented around the whole family, family-centred interventions have the potential to recognise and support Indigenous family functioning, that is, their communication, maintenance of relationships in healthy ways, decision making and problem solving [11]. Health services can also advocate to address system barriers to improved family health, such as for education, training, employment, and to child protection agencies.

There are differing definitions for family-centred healthcare, and consequently various approaches. Nixon [12] defined the delivery of family-centred care by health services as “a way of caring for children and their families within health services which ensures that care is planned around the whole family, not just the individual child/person, and in which all the family members are recognised as care recipients”. Griew, Tilton, and Stewart [13] proposed a broader two-part definition of Indigenous family-centred healthcare as: 1) movingbeyond providing care to the individual patient, to seeing them as being embedded in a family and providing services on that basis; and 2) taking a life course approach, which, without neglecting adult health, focused specific attention on establishing early life resilience and advantages through an emphasis on child development. This paper reviews the state and quality of the evidence for family-centred healthcare delivered through primaryhealthcare services for Indigenous children (from conception to 5 years). The review objectives were:

1. Outline the extent of the current evidence base for family-centred interventions by primary healthcare services for Indigenous Australian, Canadian, New Zealander or United States early childhood wellbeing;
2. Examine the conditions which enable primary healthcare services to implement family centred interventions, and the strategies they use to do so;
3. Describe the outcomes of family-centred interventions for Indigenous early childhood wellbeing.

## Methods

### Inclusion/Exclusion criteria

Studies were included in this scoping review only if they were published in English from 1 January 2000 to 31 December 2015 inclusive. The start date of the review was taken from 2000 when the US formally recognised patient-centred care as a healthcare standard [14]. Publications were also included only if the study met each of the following four criteria:

1. Participants were Indigenous Australian, Canadian, New Zealander or United States children aged from conception to five years who received family-centred care. A child was considered to be Indigenous if they were identified by the family as Indigenous (one parent may have been non-Indigenous); ‘Indigenous’ was defined using the United Nations definition of self- identification and acceptance by the community as a member [2].
2. Evaluated or described a family centred intervention or theorised a family centred healthcare model. We used Nixon’s [12] definition of family-centred healthcare *and* included: a) environmental interventions that maximise parental involvement and enhance child health or wellbeing; b) communication interventions that include parents/caregivers in collaborative care pathways, and/or reorganisation of health care to provide continuity of carers; c) educational interventions for parents/caregivers or staff; d) counselling interventions such as brief interventions, home visiting and other approaches; and/or e) family support interventions such as flexible charging schemes for poor families, referrals to other community services, parent-to-parent support [15]. We included pregnancy care models only if the intervention continued beyond the standard postpartum period of six weeks to at least three months.
3. Intervention scored greater than 26/52 points (50%) against a validated scale for family-centredness [15, 16]. The scale incorporated 13 criteria, clustered under three concepts: 1) family as a constant (family as a constant in child’s life, recognising family strengths, collaboration between parents/caregivers and professionals, needs-based family support, flexible provision of health care, sharing information with families); 2) culturally responsive (culturally competent health care, respecting family diversity, providing financial support); 3) supporting family individuality & need for different types of family support (respecting family coping methods, providing emotional support, family-to-family support, attending to the developmental needs of children and families). Each criteria were scored from zero (no evidence that the author(s) addressed, endorsed, or advocated adoption of adherence to the elements of family centred care either implicitly or explicitly) to four (numerous instances of explicit evidence that the author(s) advancedadoption or support of the elements of family-centred care).
4. Intervention was led by a primary healthcare service, defined broadly as healthcare providers involved in providing primary healthcare for Indigenous children.

### Search strategy

In consultation with an expert librarian (KK), a four-step search strategy was implemented. Step one comprised a search of 14 electronic databases: MEDLINE, PsycINFO, CINAHL, Informit, Indigenous Australia, Indigenous Studies Bibliography, AIATSIS, ATSIHealth, APAIS- ATSIS, FAMILY-ATSIS, Informit Indigenous Collection, Campbell Library, Cochrane Library, and Sociological Abstracts. MESH headings included family or parents or infant or newborn or legal guardians or pregnancy, AND child health services or Maternal Health Services or Maternal-Child Nursing or Family Health or Midwifery or Family Practice or Primary Health Care or General Practice or Delivery of Health Care or Patient-Centered Care or Health Promotion or Patient Care Planning AND Oceanic Ancestry Group OR American Native Continental Ancestry Group. Step two comprised searches of the grey literature through five clearinghouses or websites of relevant organisations in each of the four countries: Australian Indigenous Health InfoNet, Australian Institute of Family Studies, Indigenous Knowledge Network for Infant, Child and Family Health (Canada), Li Ka Shing database at St. Michael’s Hospital (Canada), and New Zealand Social Policy Evaluation and Research Unit. Search terms were: family-centred care AND children OR infant OR maternity OR trimester. Step three comprised a search of the reference lists of Indigenous maternal and child health systematic reviews. In step four, the authors of this study also drew on their knowledge of family-centred interventions.

### Identification, screening and inclusion of publications

The combined searches were imported into a bibliographic citation management software, EndNote X7 with duplicates removed. Titles and abstracts of the remaining publication titles and abstracts were screened by one author (MH). A second author (JM) retrieved and screened titles and abstracts of the remaining publications; those which did not meet inclusion criteria were excluded. The full texts of the remaining publications were retrieved and screened by blinded reviewers (RB, SC, CC, MH, JM, AR) and independent reviewers from Apunipima Cape York Health Council and Centre for Research Excellence for Improving Health Services for Aboriginal and Torres Strait Islander Children (ISAC) (KE, RM, MRM, LS, NS, KT, MW). Inconsistencies in reviewer assessments were resolved by consensus.

### Data extraction & analysis

The publications were grouped together under the name for the study. Data were extracted from the full texts for publication authorship, publication year, study design, year/s of data collection and outcome assessment interval, study setting, population and sample size. The quality of included quantitative studies was assessed by blinded reviewers (SC and CC) using the Effective Public Health Practice Project quality assessment tool [17]. Qualitative studies were assessed by blinded reviewers (MH and JM) using the Critical Appraisal Skills Programme quality assessment tool [18]. The costing study was assessed by a health economist (IK) and author (JM) using the Joanna Briggs Institute critical appraisal checklist for economic evaluations.

The publications were then imported into NVIVO software and coded (by MH). Grounded theory methods were used to map the strategies and outcomes of family-centred interventions, as well as the contexts and conditions under which they develop [19]. Grounded theory methods are well suited to conducting exploratory scoping reviews, especially in areas like family-centred interventions for Indigenous early childhood health, which is complex and has not been reviewed comprehensively before [19].

We started by coding the studies (seven publications) with the strongest study designs; then continued to code and compare the concepts in the remaining studies [19]. As we progressively coded and compared the papers, we found common or similar groups of concepts that were then recoded as higher order categories [19]. For example, across diverse studies, we identified strategies of providing subsidised fruit and vegetables; providing daily hot nutritious lunches, food coupons and hampers and nutritional supplements. We coded this concept as “augmenting diet”. As more papers were coded, similar concepts were identified, such as providing oral health products; and providing safe sleeping baskets. Consequently, we regrouped and re-categorised the earlier code as “value-adding to health through products”. Axial coding was then used to sort which of the categories represented the aim, contexts, conditions, strategies and outcomes of the family- centred interventions and to identify the interrelations between these [20]. Through axial coding, for example, “value-adding to health through products” became part of a core strategy titled “supporting family behaviours and self-care”. These analytic coding steps did not occur in a lineal order as described here, but were performed interactively, revisiting and refining concepts and categories as new insights occurred [19].

## Results

**Fig. 1:**
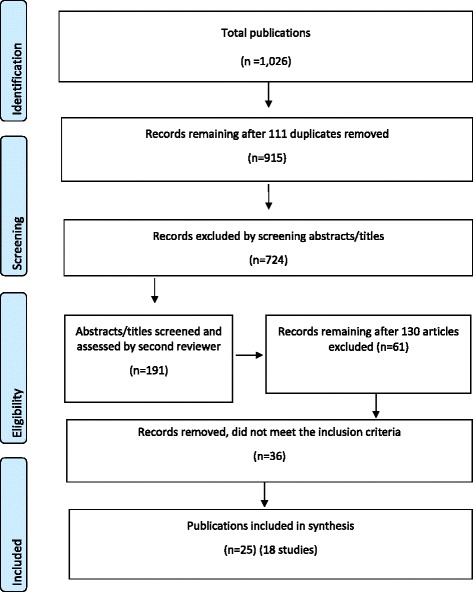
Flowchart of publications included in the review

A Preferred Reporting Items for Systematic Reviews and Meta-Analyses (PRISMA) flowchart is presented at Fig. 1 [21]. The process of identification, screening and inclusion of publications resulted in 18 included studies (25 publications). One study of the US Family Spirit intervention was reported in five publications [22–26]; the Australian Baby Basket program in three publications [27–29], the Australian Triple P parenting study intwo publications [30, 31]; and the remaining studies had one publication each.

### Characteristics of studies

Thirteen of the included 25 publications (52%) were published in the last four years (2012–2015). Eleven of the 18 studies were Australian (61%); three Canadian (17%); one from New Zealand (6%); and three from the USA (17%). Only 13/18 studies stated their setting; these being spread fairly evenly across urban (5/13 or 38%), rural (4/13 or 31%) and remote areas (4/13 or 31%) (Table 1).

**Table 1:** Characteristics of studies

Study	First author year	Country setting	Participants	Aim of study	Details of study	Study quality	Implementation strategies	Outcomes
1	Abel 2015 [32]	NZ rural	12 Maori mothers and 10 key community stakeholders	Understand determining factors for the acceptability of the Wahakura as an infant sleeping device	Exploratory qualitative study; Interviews either at home or work; Ethics approval	Moderate	Simple, woven flax bassinet-like structure to be used in parental bed; ‘safe sleeping rules’ aimed at reducing sudden unexpected death	Practical value (safe bed-sharing, easier breast feeding, portability, versatility, convenience); cultural and spiritual value (natural fibre, sacred and healing qualities); health promotion (the process of weaving resulted in some women giving up smoking)
2	Applequist 2000 [43]	USA	52 Native American female caregivers of children with a disability recruited from three early intervention programs	Determine parental satisfaction with services	Qualitative evaluation; One time-point interviews; No ethics approval reported	Moderate	Educational and therapeutic services provided in home-based, clinical or centre-based settings, primarily by paraprofessionals	Caregivers were generally satisfied; more so with early intervention programs perceived as more family-centred. Satisfaction not correlated with provider nor family variables
3	Arney 2010 [46]	AU urban	Mothers, fathers and extended family members who supported a family member in the program 60 participants recruited by program nurses and cultural consultants	To explore Aboriginal families’ perceptions on the ‘Family Home Visiting Program’ in Adelaide SA	Qualitative evaluation; One time-point focus groups and interviews; No ethics approval reported	Weak	Home-based intervention delivered by Child and Family Health Nurses, and Cultural Consultants/ Aboriginal staff. Intensive staff training in strength- based approach, attachment, child development, and socio- emotional issues	Families valued family inclusiveness, cultural respect, strengths- based approach, flexibility to address family-identified issues, program convenience (home delivery) and Aboriginal staff as a bridge with the mainstream service
4	Atkinson 2001 [33]	AU urban	Representatives from maternal and child health services in the Indigenous community	To describe the development of a new Maternal and Child Health Program run by the Townsville Aboriginal Health Service	Qualitative exploratory study; Single time-point forum focussed on quality improvement, held August 1999; No ethics approval reported	Weak	Daily maternal and child health care plus primary health care through collaborative approach with hospital, university, health service, and Centrelink. Breast feeding, nutrition, and smoking cessation program. Child friendly waiting room	Increased ante-natal visits; decreased pre- term births, low birth weight, and peri-natal deaths. Need for: team approach for Indigenous mothers and infants; improved coordination of services; improved transport and education
5	Ball 2005 [34]	CAN rural	First Nations community members from three communities	To report on promising practices of integrated service models centred around early childhood care and development programs through a community development approach	Series of group forums and individual interviews; No ethics approval reported	Weak	Multi-purpose centre at public school: child care, parent education and support; service referral; Nutritious meals; preventive dental care; Primary health care incl. immunisation, vision, hearing, and speech screening; Specialist services incl. support for children with foetal alcohol spectrum disorder; speech therapy; Cultural activities; Social services; Community kitchen and gathering space. Training program in child and youth care	Service centres can become a focal point for mobilising community involvement in supporting young children and families; social cohesion; a cultural frame around service usage to inform external service providers and offer cultural safety for community members
6	Barlow 2015 [23]	USA rural	Pregnant American Indian teens 322 participants recruited from Indian health service clinics; women, infants, and children nutrition programs; schools, and by word of mouth Intervention Group *n* = 159; Control Group *n* = 163	To assess the efficacy of the ‘Family Spirit’ intervention for parenting, and for maternal and child emotional and behavioural outcomes	Randomised controlled trial (RCT) – Family Spirit intervention plus optimised care compared with optimised care only; outcomes assessed at baseline (28 to 32 weeks of gestation), 36 weeks of gestation; and 2, 6, 12, 18, 24, 30, and 36 months postpartum through maternal self-report questionnaires, in-person interviews, audio computer-assisted self-interviews, observational data, and medical chart data; Ethics approval	Strong	43 structured pre-natal and infant care lessons in “positive parenting” addressing maternal behaviour and mental health problems; delivered in participant’s homes by American Indian paraprofessional health educators; Educators received > 500 h training	Parents: Increased parenting knowledge and locus of control; fewer depressive symptoms, and externalising problems; lower use of marijuana and illegal drugs Children: Fewer externalising, internalising, and dysregulation problems
	Barlow 2013 [22]			To assess parenting and maternal and early child behavioural outcomes from pregnancy to 12 months postpartum	Outcomes assessed at baseline (32 weeks’ gestation) and 2, 6, 12 months postpartum			As above. Increased home safety attitudes
	Mullany 2012 [25]			Describes rationale, design, methods, and baseline results of the Family Spirit intervention	Community-based participatory research In January 2007, eligibility criteria – minimum gestational age was increased to 32 weeks			Moderate to high scores in maternal psychological and behavioural risks; higher lifetime cigarette use
	Walkup 2009 [26]		167 participants recruited from pre-natal, and school-based clinics, between May 2002 and May 2004. Intervention Group *n* = 81; Control Group *n* = 86	15 months’ pilot trial	Outcomes assessed at baseline (28 weeks’ gestation); 2, 6, and 12 months postpartum. Follow up completed in May 2005.		25 home visits/1 h each. Breastfeeding Nutrition program: 23 home visits/1 h each	Mothers reported increased parenting knowledge Decreased infantile externalising behaviour and separation distress
	Barlow 2006 [24]		53 participants recruited between July 2001 and Feb 2002 from four American Indian health service catchments; Intervention Group *n* = 28; Control Group *n* = 25	9 months’ pilot trial	Follow-up data available for only 19 intervention and 22 control participants		Breastfeeding education program only; 25 home visits and 41 discrete lessons provided from 28 weeks’ gestation to 6 months postpartum	Increased parenting knowledge, skills, and involvement. Mothers in the intervention group experienced a larger drop in depressive symptoms.
7	Black 2013 [35]	AU rural	167 disadvantaged Aboriginal children, aged 0–17 years with nutrition risk identified and recruited by Medical services staff	To evaluate the impact of a fruit and vegetable subsidy program, delivered by an Aboriginal Medical Service, on short-term health outcomes	Uncontrolled before & after study; Outcomes measures assessed after 12 months; Clinical assessments, health record audits and blood testing; Ethics approval	Weak	Provision of a weekly box of subsidised fruit and vegetables linked to preventative health services and nutrition promotion	Fewer visits to health services, hospital emergency department attendances, and prescription in oral antibiotics. A small but significant increase in mean haemoglobin levels but no change in the proportion with iron deficiency and anaemia
8	Blinkhorn 2012 [36]	AU	Aboriginal Health workers from six health services will recruit 72 families with a child six months of age	To monitor a longitudinal oral health education program to assess the effect on dental caries, feasibility, and to inform the design of a confirmatory randomised phase three trial	Study protocol - longitudinal study Repeated measures over 2 years on parental knowledge and views on acceptability of the program; Data on dental caries will be compared with data from a historical reference group; Ethics approval	N/A	Aboriginal Health Workers (AHWs) will provide advice on diet, oral health products, child specific dental advice, education material, and screening for early childhood caries; invite mothers to ACCHS clinic; home visits if appointments missed or difficulties attending clinic	N/A
9	D’Espaignet 2003 [37]	AU remote	Aboriginal pregnant women; 7730 hospital-based live births between 1988 and 2001 analysed	To assess the effect of ‘Strong Women, Strong Babies, Strong Culture’ health education program on birth weights	Controlled before and after study; Group 1 commenced program in 1993; Group 2 in 1996 and 1997; Ethics approval	Weak/Moderate	Senior Aboriginal women provided advice and encouragement about healthy pregnancy management in relation to nutrition (including bush foods), safe practices such as alcohol and smoking abstinence, reinforcing the need to seek adequate and timely medical help and to take prescribed medicines	Significant improvements in birthweight in Group 1, but no significant change in Group 2; Ante-natal care aspects could not be assessed due to incomplete electronic data collection
10	Di Lallo 2014 [44]	CAN	First Nations pregnant women 281 women attended the program between November 2005 and February 2009	Evaluate the Aboriginal Prenatal Wellness Program	Program evaluation Pre and post survey on participant satisfaction No ethics approval reported	Weak	Service provided on a continuum of care involving community agencies, health professionals, social workers, life support counsellor and Aboriginal community Elders	General high satisfaction. Improved access to ante-natal health care that is culturally sensitive, inclusive, efficient and supportive. Increase in returning clientele. Increased breast feeding. Decreased maternal smoking and drinking
11	Edmunds, 2016	AUS remote	170 Aboriginal pregnant women and mothers and babies to 6 months post- partum from Cape York communities, Aboriginal Health Workers	Evaluate the impact of the Baby Basket program as implemented in Cape York by Apunipima Cape York Health Council, and aspects of the program that are transferable to other regions and other groups	Mixed method study: qualitative grounded theory methods based on interviews and focus groups with women who received Baby Baskets, family members, and healthcare workers. Quantitative comparative analysis of routine indicators of Apunipima communities and nearby Gulf and Torres; and Baby Basket surveys. Cost analysis to estimate the resources required to deliver the Baby Basket	Costing: Moderate Qualitative: Moderate Quantitative: Weak	Encourages early and frequent attendance at antenatal clinics and regular postnatal check- ups. Engagement is facilitated by delivery of three Baby Baskets including five food vouchers to women. Baskets are delivered in the first trimester, immediately prior to birth and post birth. Education about healthy choices around smoking, alcohol and diet.	The core concern of implementation was termed working towards an empowering family- centred approach. Compared with the control sites: Apunipima sites had a higher proportion of early and more frequent antenatal visits, lower levels of iron deficiency in pregnant women, declining levels of faltering growth in children. But also increasing smoking in pregnant women and inconsistent results about education. Cost per participant was modest ($874).
	McCalman, 2015 [29]							
	McCalman, 2014 [28]							
11	Elliott 2012 [38]	AU remote	Aboriginal parents and carers of all children born in the Fitzroy Valley region, Western Australia between 2002 and 2003	Describe strategy development for the diagnosis and prevention of Foetal Alcohol Spectrum Disorders (FASD); and the support for parents and carers of affected children through individual treatment plans	Descriptive study Information about antenatal exposures; early life trauma; and health and development of parents and carers was obtained via a medical checklist; Ethics approval	N/A	Aboriginal organisations partnered with researchers to successfully lobby for restricted access to alcohol; conducted a FASD prevalence study following extensive community consultation and consent. Program includes community education; support for parents and carers; advice for teachers	Data will be used by the community to advocate for improved services and new models of health care
12	Harvey- Berino 2003 [39]	USA	43 mothers and their preschool Native- American children	To determine whether maternal participation in an obesity prevention plus parenting support program was feasible and effective in reducing the prevalence of childhood obesity	RCT comparing obesity prevention & parenting support with parenting support alone; 40 participants assessed; 20 each in treatment and control groups; Recruitment via media advertisements, day care centres, nutrition program, self-referral, informal networking in community; Outcome measures assessed at baseline and 16 weeks; Ethics approval	Moderate	11 parenting lessons conducted over 16 weeks in the parent’s home; training provided for peer educator and project director	Decreased weight gain in children in the obesity prevention & parenting support group. Inconclusive data on whether parents posing restrictions on feeding influenced weight gain
13	Homer 2012 [40]	AU urban	353 Aboriginal and Torres Strait Islander pregnant women who attended the Malabar service and gave birth during 2007 and 2008	To evaluate whether and to what extent the Malabar Community Midwifery Link Service was meeting the needs of women clients and staff	Before and after study; Repeated measures of clinical data and data on smoking/alcohol use; Focus-group data at one time-point of womens’ satisfaction with the service; Ethics approval	Moderate (qualitative component); Weak (quantitative component)	Midwifery continuity of care during pregnancy, labour and birth; and post-natally with referral to child health services after discharge; service is either hospital or home based; transport provided for better access	Women felt the service provided ease of access, continuity of care and carer, trust and trusting relationships. Early access to pregnancy care. Reduced smoking during pregnancy. Health promotion programs developed that target smoking and alcohol consumption during pregnancy
14	Munns 2010 [41]	AU remote	Parents, carers, and ante-natal clients of children aged 0–3 years living in the town of Halls Creek, Western Australia	To describe the introduction of an Indigenous home visiting parent support program to enhance promotion of behavioural and attitudinal changes to parenting	Case study/program description; A group of strong men and women as home visitors; working in conjunction with community child health nurses and midwives; No ethics approval reported	N/A	Enhanced promotion of behavioural and attitudinal changes to parenting; monthly 1 h home visits by Indigenous peer support team (extended and in other locations if needed); may be two or three home visitors to accommodate different languages, family, and cultural issues; health promotion through pictorial handouts; Inclusion of culture and lore. Train the trainer program	Not reported
15	Poole 2000 [42]	CAN urban	18 pregnant Aboriginal women with substance use problems who accessed the service in 1988; tracking of 12 clients who accessed services July 1999 and December 1999; surveys completed by 10 staff and three Council members; survey completed by 21 key informants	Evaluation of the Sheway Program	Qualitative program evaluation; Art expression combined with a focus group to capture women’s perspectives on the service. File review of birth and health outcomes. Data compared with information on women clients from two previous years. No ethics approval reported	Moderate	Daily hot nutritious lunches, food coupons, food bank hampers and nutritional supplements, bus fare for appointments, formula, nappies, clothing, equipment and other items for newborn infants, outreach and home visits, recreational and creative programs, nutrition counselling and support, alcohol and drug counselling, methadone prescribing, support in developing/ improving parenting skills, advocacy on housing and legal issues	Improved nutritional outcomes, decreased substance misuse, improvement in housing, lower rates of child apprehension by the Ministry of Children and Family development, healthier birth weights, up-to date immunisations
16	Thomas 2015 [45]	AU	12 service managers, paediatric registrars, early childhood health nurse, midwife, Aboriginal health education officer, speech pathologist, manager of parenting support program	To explore the views of service providers on how paediatric outreach services work in partnership with other services, Aboriginal families and the community, and how those partnerships could be improved	Qualitative one-point in time study; In-depth semi- structured interviews, focus groups; Ethics approval	Moderate	Formal and informal approaches to facilitate relationships between service providers and families, ensuring children receive quality care when and where they need it. Partnerships founded on a culturally appropriate model of care that was non-judgemental, based on trust and respect, and recognised holistic health and wellness	More time for consultations and more opportunity for follow-up than would normally occur in the outpatient setting; leadership was essential component of effective partnerships
17	Turner 2007a [30]	AU urban	51 Indigenous families; *n* = 26 treatment group, *n* = 25 control group (waitlist for 8 weeks)	To assess the effectiveness and cultural appropriateness of the Triple P parenting program	Randomised group design with repeated measures; outcome measures assessed at 6 months; recruitment through home-based interview; no ethics approval reported	Moderate	Eight-session program, using active skills training process to help parents acquire new knowledge and skills.	High consumer satisfaction; break down of obstacles in accessing mainstream services; significant decreases in problem child behaviour; significant decrease in reliance on dysfunctional parenting practices
	Turner 2007b [31]		Non-Indigenous researchers	To reflect on a culturally sensitive adaptation of a mainstream intervention, the “Triple P Parenting Program”	Reflective paper No ethics approval reported	N/A		Appointing project staff can be complex and sensitive. Need community acceptance and support; sensitivity to participant’s issues; flexible access to services; strategies to overcome literacy and language barriers; awareness that complex family issues may impact group dynamics; sharing of outcomes with community

Twelve/18 studies reported more than one study population. The majority of studies targeted expectant women or new mothers. In order of frequency, other client groups were: Indigenous children, parents/caregivers and other family members and other community members and stakeholders. Program deliverers, in order of frequency, were: Indigenous health paraprofessionals/workers, senior/Elder women who provided education or support, other health practitioners, senior/Elder men, and partnerships withresearchers. This diversity was related to the inclusivity of many family-centred approaches and the varied modes of their delivery.

### Study design

**Fig. 2:**
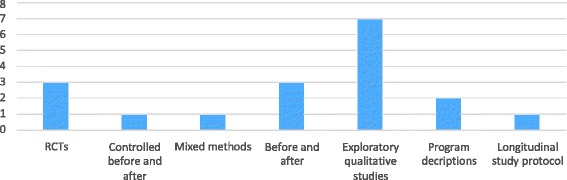
Number of each type of study design

There were three/18 randomised controlled studies (17%), one controlled before and after study (5%) and one mixed method evaluation (5%) to test the impact of family-centred interventions on the quality and effectiveness of care. However, the remaining 13/18 studies (72%) were non-comparison studies, including three uncontrolled before and after studies, seven exploratory qualitative studies two program descriptions and a protocol for a longitudinal study (Fig. 2).

### Study quality

Only one/18 studies was rated of strong quality [22–26] (Table 1). This study randomised 322 participants to the US Family Spirit intervention or optimised standard care, and evaluated outcomes at different time points using validated measurement tools. The other assessable studies were of moderate (7/18) moderate/weak (1/18), or weak (6/18) quality, with lack of consistently strong methodology across the majority of assessed criteria. The quality of two program descriptions and one study protocol were not assessed.

### Key elements of family-centred interventions

The aims, strategies, conditions and outcomes of family-centred care reported in each study are summarised in Table 2, where ✓ denotes evidence that the author(s) advanced adoption or support of the element of family-centred care, ~ denotes an implicit or inferred reference consistent with the intent of that element; and X denotes no evidence for that element of family-centred care.

**Table 2:** Intervention aims, strategies, enablers andoutcomes

	Aim	Strategies						Enabling conditions				Outcomes							
First author year	Promote healthy families	Supporting family behaviours & self-care	Increasing maternal knowledge and skills	Linking with the clinic	Building the Indigenous workforce	Promoting cultural/community connectedness	Advocating for social determinants of health	Competent and compassionate staff	Flexibility of access	Continuity & integration of care	Culturally supportive care	Child nutritional status	Child emotional behaviour	Child preventive health incl. safety	Parental depression, substance use	Parenting knowledge, confidence and skills	Health service satisfaction	Health service utilisation/access and cost	Community/cultural reviatlisation
Abel, 2015 [33]	✓	✓	✓	X	~	✓	X	X	✓	X	✓	X	X	✓	✓	✓	✓	X	✓
Applequist, 2000 [44]	~	✓	✓	~	✓	X	X	✓	✓	✓	✓	X	✓	X	X	✓	✓	X	X
Arney, 2010 [47]	~	✓	✓	~	✓	X	~	✓	✓	✓	✓	X	X	~	~	✓	✓	X	X
Atkinson, 2001 [34]	✓	X	✓	✓	✓	X	X	~	✓	✓	✓	✓	X	X	X	~	✓	✓	X
Ball, 2005 [35]	✓	✓	✓	✓	✓	✓	✓	~	✓	✓	✓	~	✓	✓	~	✓	✓	X	✓
Barlow, 2015 [24], 2013 [23], 2006 [25]; Mullany 2012 [26], Walkup, 2009 [27]	✓	~	✓	~	✓	X	X	✓	✓	✓	✓	X	✓	✓	✓	✓	X	X	✓
Black, 2013 [36]	✓	✓	✓	~	X	X	X	~	✓	✓	~	✓	X	✓	X	~	X	✓	✓
Blinkhorn, 2012 [37]	✓	✓	✓	~	✓	X	X	X	✓	✓	✓	X	X	X	X	X	X	X	X
D’Espaignet, 2003 [38]	✓	✓	✓	✓	✓	✓	X	✓	✓	X	✓	✓	X	X	X	~	X	X	✓
Di Lallo, 2014 [45]	~	✓	✓	✓	✓	X	~	✓	✓	✓	✓	✓	X	X	✓	~	✓	✓	X
Elliott, 2012 [39]	✓	✓	✓	~	✓	✓	✓	X	✓	X	✓	X	X	X	X	X	X	X	~
Harvey-Berino, 2003 [40]	✓	✓	✓	~	✓	X	X	X	✓	X	~	✓	X	X	X	✓	X	X	X
Homer, 2012 [41]	✓	X	✓	✓	✓	X	X	✓	✓	✓	✓	X	X	X	✓	~	✓	✓	X
McCalman, 2014 [29], 2015 [30]; Edmunds 2016 [54]	✓	✓	✓	✓	✓	X	X	✓	✓	~	✓	✓	X	X	✓	✓	✓	✓	X
Munns, 2010 [42]	✓	✓	✓	~	✓	X	X	✓	✓	✓	✓	X	X	X	X	X	~	X	X
Poole, 2000 [43]	✓	✓	✓	~	✓	✓	✓	✓	✓	✓	✓	✓	X	✓	✓	~	✓	X	X
Thomas, 2015 [46]	~	X	✓	✓	X	X	X	✓	✓	✓	✓	X	X	X	X	X	~	✓	X
Turner, 2007a [31]; 2007b [32]	✓	✓	✓	✓	~	X	X	✓	✓	X	✓	X	✓	X	X	✓	✓	✓	X

### Aim of family-centred interventions

The aim of study interventions was to promote healthy families; that is, to enable families to increase control over and to improve their health. In 14/18 studies (78%), this aim was explicitly reported [22–42], and in the other four, it was inferred (Table 2). Examples of an explicit aim were to assess the effectiveness and cultural appropriateness of the Triple P parenting program [31]; and to evaluate the impact of a weekly subsidised box of fruit and vegetables [35]. Examples of an inferred aim were to determine family satisfaction with a family-centred service [43, 44] and to explore the views of service providers about how family-centred services work [45].

### Strategies of family-centred interventions

Six key strategies were identified: supporting family behaviours and self-care, increasing maternal knowledge, linking with the clinic, building the Indigenous workforce, promoting cultural/ community connectedness and advocating for social determinants of health (Table 2). Intervention components varied, with many having multiple strategies.

#### Supporting healthy family behaviours and self-care

Fourteen studies (78%) explicitly described or evaluated the provision of mentoring, counselling, advocacy and products to support healthy family behaviours and self-care [28–34, 41–44, 46]. Mentoring by Indigenous Elders and/or health professionals was provided to encourage reduced or no alcohol use and smoking in pregnancy [28, 29, 37, 38, 42, 43]; improve nutrition in pregnancy [28, 29, 35, 37, 42, 44]; safe sleeping [28, 29, 32]; early childhood healthy eating and exercise routines to reduce obesity [28, 29, 39]; and care for and learning by disabled children [40]. As well, parents/caregivers were mentored to care for themselves [43] and reward themselves for meeting goals [36]. Counselling or brief interventions were provided to enhance nutrition and reduce alcohol and drug use [28, 29, 42]. Advocacy was also reported, for example to assist with housing, welfare and legal issues [28, 29, 42] and for improved services and new models of healthcare [38].

Products, such as food and nutritional supplements, were provided to support women during pregnancy. For example, the Australian Baby Basket program provided antenatal, perinatal and postnatal baskets to Cape York women, which included a baby bed, educational books and clothing, nappies and other items for the baby and mother [29]. The Canadian Sheway program provided daily hot nutritious lunches, food coupons, food bank hampers and nutritional supplements for pregnant women struggling with substance abuse and addictions [42]. Products were also provided for new born infants, such as formula, nappies, clothing, and equipment such as sleeping baskets. Examples included the New Zealand Wahakura, a flax bassinet which was provided to promote safe sleeping for Maori infants [29], the Canadian Sheway program’s provision of items for newborn infants [42], and an Australian Aboriginal Medical Service’s provision of a weekly box of subsidised fruit and vegetables linked to preventative health services and nutrition promotion [35].

#### Increasing maternal knowledge and skills

All 18 studies (100%) explicitly evaluated or described maternal health education and skills development. The foci of these strategies was to promote maternal skills generally, e.g. [22–26, 28–30], problem solving and coping skills [26, 31], goal setting [24, 46], breast feeding and nutrition skills [28, 29, 39], dental health knowledge [36], safe sleeping [28, 29], smoking and alcohol reduction [28, 29] and the promotion of children’s competence and development and management of misbehaviour [31]. Group or individual parent education was delivered in formal training or in home settings. To overcome literacy and language barriers, training was provided in intensive small group sessions or individually [26, 30, 31], and resources were made available in simple English, audio visually, and as table top flip charts [26, 28, 29, 31].

#### Linking with the clinic

Eight studies (44%) explicitly reported linking families with clinical services [27, 28, 30, 31, 34, 37, 41, 42]. In some interventions, program educators encouraged family members to attend the health clinic for antenatal checks and birthing [28, 29, 33, 40, 44], to seek timely medical help [31, 37], for immunization [28, 29, 34, 40], screening for vision, hearing and speech [34], and specialist paediatric services [34, 45].

#### Building the Indigenous workforce

Fourteen studies (78%) reported employment, training and supervision of an Indigenous workforce as a strategy [22–26, 28, 29, 33–46]. For example, two newly graduated Aboriginal midwives were mentored through an urban Australian community midwifery service [40].

The Native American educators of the Family Spirit intervention were required to complete 500 h of training in home-visiting methods and curricular content, had to demonstrate competency in the form of written and oral examinations, and received daily on-site supervision and weekly cross-site conference calls [22–26]. Similarly, an Australian nurse home-visiting intervention provided extensive training for Aboriginal staff instrength-based approaches to attachment theory, child development and socio-emotional issues facing families [28, 29, 46].

#### Promoting cultural/Community connectedness

Five studies (28%) explicitly promoted cultural, spiritual or community connectedness as a strategy [32, 34, 37, 41, 42]. For example, new Maori parents were encouraged to use a safe sleeping device made from native flax, a material which had traditionally been used for weaving and was considered to have sacred and healing qualities [32]. Aboriginal Australian pregnant women were encouraged to make greater use of bush foods [37] and to become more engaged with local community events [41]. Canadian studies described early childhood care and development programs as a ‘hub’ for meeting a range of service and social support needs of community members [34] and encouraged pregnant women to identify a network of people whom they could call upon for support [42].

#### Advocating for social determinants of health

Three studies (17%) described advocacy to improve aspects of the social and/or economic determinants of health [34, 38, 42]. Studies considered family-centred care to be a ‘hook’ for mobilising community involvement in supporting young children and families [34], advocated to restrict the sale of full-strength alcohol [38], and provided advocacy and support for child access and custody, other legal issues and housing [42].

### Enablers of family-centred interventions

The four enablers of family-centred interventions were competent and compassionate program deliverers, flexibility of access, continuity and integration of care, and culturally supportive care (Table 2).

#### Competent and compassionate program deliverers

Eleven studies (61%) cited the importance of having competent and compassionate staff as an enabler of family-centred care [22–26, 28–31, 37, 40–44, 46]. For example, Arney et al. [46] found that families’ views about the program could not be separated from their appreciation of the qualities and abilities of the staff. Barlow et al. [22–24] required staff to have the ability to show compassion, be non-judgmental and have inter-personal skills.

Other publications emphasised the need for cultural sensitivity training to promote the interaction of practitioners with clients in ways that respected their cultural orientations and living situations [26, 30, 31]. Seven publications referred to the cultural competence of Indigenous program deliverers who could accommodate different languages, family and cultural issues [22–24, 28, 29, 37, 41, 42]. Homer et al. [40] however, found that it was the trusting relationship between provider and client that was important; this was not necessarily with an Aboriginal provider. Applequist & Bailey [43] found that 96% clients indicated no preference regarding the ethnic background of their service provider.

#### Flexibility of access

Another hallmark of family-centred care interventions was the flexibility of access provided to health education and care. All 18 of the included studies (100%) reported flexibility of access, including the provision of home-based care, e.g. [22–26, 28, 29, 31], choice of training location, e.g. [30, 31], or less commonly, the provision of transport or transport vouchers to and from services [40, 42]. Service providers considered it important to provide flexible access as an enabler of engagement, particularly to families without means of transport.

#### Continuity and integration of healthcare

Another enabler, reported in 12/18 studies (67%), was the provision of healthcare continuity and integration by linking women across antenatal, birthing and postnatal services and providing integrated wrap-around care [22–26, 33–36, 40–46]. For example, Homer et al. [40] described a healthcare model whereby women were offered continuity of midwifery care during pregnancy, labour and birth; and referral to child health services post- natally after discharge.

Community agencies, health professionals, social workers, life support counsellors, and community Elders collaborated to provide integrated, wrap-around care for families [41, 44, 46]. Intercultural collaboration across Indigenous and mainstream health services was also considered important [44–46]. Leadership was considered an essential component of effective partnerships with other services, families and the community as it enhanced workplace ethos and created an environment where collaboration was supported [45].

#### Culturally supportive care

Culturally supportive care, based on secure, respectful and reciprocal relationships and partnerships with explicit respect for diversity, was highlighted in 16/18 studies (89%) [22–26, 28–38, 40–46]. Being community driven, e.g. [38] or incorporating culture and lore, e.g. [41] was seen to enhance the effectiveness of programs and break down obstacles to accessing mainstream services, e.g. [31]. In some interventions, clients were provided a choice of the participants’ native language or English for health education delivery [22–26, 41].

### Outcomes

Intervention outcomes were reported in the 15/18 evaluation studies (83%) [22–26, 28, 29, 31–34, 37, 39, 40, 42–46] for Indigenous children, parents/caregivers, health services, and broader community/culture (Table 2). For Indigenous children, reported outcomes included improved nutritional status, emotional and behavioural and preventive health. For parents/caregivers of Indigenous children, studies reported reduced parental/caregiver depression and substance abuse, and improved parenting/caregiving knowledge, confidence and skills. For health services, reported outcomes included client satisfaction and improved service utilisation and cost of delivery. Community/cultural revitalisation was also reported. Two studies that described programs [38, 41] and one study protocol [36] did not report outcomes.

### Child health outcomes

#### Children’s nutritional status

Seven/15 studies (47%) reported improvements in children’s nutritional status including changes in weight (overweight and underweight), growth and/or nutritional markers such as increased haemoglobin levels [28, 29, 33, 35, 37, 39, 42, 44]. Improved birth weights were reported following advice in relation to nutrition, alcohol and smoking during pregnancy, and utilisation of adequate and timely medical help [33, 37, 42]. Increased breast feeding was reported in a self-report survey following an Aboriginal Prenatal Wellness Program [44]. A reduced incidence of faltering growth was reported in an evaluation of the Australian Baby Basket program [28] and a small but significant increase in mean haemoglobin levels of children was found in a similar Australian study following the provision of a weekly box of subsidised fruit and vegetables linked to preventative health services and nutrition promotion [35]. Finally, decreased weight gain in children in the obesity prevention group of a US randomised controlled trial was found following an obesity prevention intervention with mothers of preschool Native-American children [39].

#### Children’s emotional behaviour

Four/15 studies (27%) reported improvements in children’s emotional behavior [22–26, 31, 34, 43]. Improved coping strategies, self-expression and compliance were reported, as were lower rates of infant separation distress and child anxiety [22, 23, 26, 31, 42]. Fewer behavioural problems such as physical aggression, disobeying rules, fearfulness, separation distress, social withdrawal, or poorly modulated emotional responses in children were also found in the US ‘Family Spirit’ [22, 23, 26] and Australian Triple P [31] interventions.

#### Preventing childhood injury and illness

Five/15 studies (33%) reported outcomes related to the prevention of childhood injury and illness [22–26, 32, 34, 35]. Improvements were found in attitudes toward, or actual home safety [22, 23, 32, 35]. For example, the US Family Spirit intervention resulted in an increased awareness of home safety issues in teen mothers [22, 23, 26]. The New Zealand Wahakura, a woven flax bassinet delivered with safe sleep messages, improved parental reassurance and confidence while providing the infant with a safe place to sleep in the parental bed [32]. The Canadian Sheway program resulted in housing improvement and lower rates of child apprehension by the Ministry of Children and Family development [42]. Studies also reported up to date immunisations [34, 42], screening for children’s vision, hearing, and speech [34], and a significant decrease in prescribed oral antibiotics [35].

### Parent/carer outcomes

#### Parent/Carer’s depression and substance misuse

Six/15 studies (40%) reported reductions in parental/carer depression and/or substance misuse [22–24, 26, 28, 29, 32, 40, 42, 44]. For example, American Indian teen mothers had fewer externalising problems and depressive symptoms after participation in the Family Spirit intervention [22, 23]. Similarly, Poole [42] reported decreased substance misuse by pregnant women who participated in the Canadian Sheway program. Also reported were reductions in maternal smoking [32, 40, 42, 44] and use of marijuana and other illegal drugs [23, 42]. The Australian Baby Basket program was associated with a decrease in women who consumed alcohol during pregnancy over time. All women who consumed alcohol during pregnancy in 2013 were provided a brief intervention [28].

#### Parenting/Caregiving knowledge, confidence and skills

Eight/15 studies (53%) reported improvements in parenting/caregiving knowledge, confidence and skills [22–24, 26, 28, 29, 31, 32, 39, 43, 46]. For example, improved parenting knowledge and locus of control were found in Native American teen mothers following the US Family Spirit intervention [22–24, 26]. Similarly, an Australian nurse- delivered home visiting program resulted in an improved sense of confidence in parenting [46]. Turner et al. [31] and Munns [41] found behavioural and attitudinal changes to parenting including a significant decrease in reliance on some dysfunctional parenting skills. The other five publications that explicitly aimed to enhance parental skills and practices were protocols or program descriptions and did not report outcomes.

### Health service outcomes

#### Satisfaction with healthcare

Ten/15 studies (67%) reported high satisfaction with family-centred health service provision [28, 29, 31–35, 40, 42–44, 46] with greater satisfaction reported for programs that were perceived to be more family-centred [43].

#### Healthcare access, utilisation and cost

Seven/15 studies (47%) reported improved health access or utilisation as an outcome of family-centred care [24, 27–29, 31, 33, 35, 40, 44, 45]. Culturally appropriate services were seen to promote more time for consultations and more opportunity for follow-up than would normally occur in an outpatient setting [28, 44, 45]. Also reported were earlier and increased utilisation of ante-natal care services [28, 33, 40] and a breakdown of some of the obstacles Indigenous families faced in accessing mainstream services [31]. A reduction in visits to health services for illness, hospital emergency department attendances and oral antibiotic prescriptions was also found [35]. The Australian Baby Basket program evaluation reported that the cost per Baby Basket participant of about $874 appeared to be a modest investment to provide babies with a better start in life [27].

### Community/cultural revitalization

Finally, five/15 studies (33%) reported community or cultural revitalisation as a result of implementing a family-centred intervention [22–24, 26, 32, 34, 35, 37]. The cultural and spiritual value of interventions was considered to be an outcome in its own right; for example, the Wahakura woven flax bassinet had cultural and spiritual value as well as promoting safe sleeping practices [32]. Centre-based interventions also became a focal hub for mobilising community involvement in supporting young children and families and encouraging social cohesion [34], as well as a basis to advocate for improved models of healthcare that offered cultural safety for community members [34, 38]. The employment of Indigenous para-professionals was also considered to have the potential to break multigenerational cycles of behavioural health disparities for Indigenous communities [22, 23, 26, 35, 37].

### Limitations

Although a rigorous and thorough search strategy was used, it is possible that this scoping review did not locate all relevant studies. There was high level of agreement between blinded coders, and consensus on all included studies, but it is also possible that relevant intervention descriptions or evaluations may have been misclassified. Since evaluations with statistically significant findings are more likely to be published, it is possible that the published evaluations reviewed overestimate the true effectiveness of family-centred interventions in health care for Indigenous peoples [47].

## Discussion

This review considered the current evidence base for the impact of family-centred interventions on Indigenous early childhood health. Like other reviews of Indigenous health [48, 49], we found little impact evaluation research that aimed to test the effectiveness of interventions, and only one study was rated of strong quality. The preponderance of the literature about family-centred interventions focussed on program descriptions or qualitative process evaluations, which explore the concepts and issues and described the interventions and formative or intermediate outcomes. It is likely that this is because the field is still in the relatively early stages of development, therefore there has not been enough elapsed time for follow-up studies and thus we do not know the full impact on Indigenous families of family-centred interventions.

The best evidence available suggest family-centred interventions can not only improve Indigenous children’s health but also the health of their parents/caregivers. Studies suggest that outcomes include improved birth weights [33, 37, 42] and reduced weight gain of obese children [39], reduced children’s problem behaviours [22, 23, 26, 31], improved home safety, e.g. [23, 32, 42], and improved immunisation and screening rates [34, 35, 42].

Interventions also increased parenting knowledge [22, 24, 26, 31], involvement [24], locus of control [23], self-efficacy [22] and decreased reliance on some dysfunctional parenting practices [31]. Through improving parenting knowledge and skills, the interventions may have reduced the physical aggression of parents/caregivers [22, 23], depressive symptoms and past month use of marijuana and illegal drugs [23]. Health services experienced high rates of consumer satisfaction [31, 43], and improved access to mainstream services [31]. No adverse effects were reported. No study directly addressed the ultimate outcome of decreased morbidity as a result of the intervention.

A key gap in the evidence related to family engagement with and positioning in interventions. Family-centred care is based on the principle that parents bring expertise at both the individual care-giving level and the systems level [50]. However, few studies reported the extent to which families engaged in the family-centred interventions. Instead studies described the intervention components of a family-centred approach, focussed on their acceptability or feasibility, or users’ satisfaction with services, or evaluated their health outcomes and/or costs. Thus MacKean’s ([50] p. 81) observation of mainstream healthcare settings where; “family-centred care is beginning to sound like something that is being defined by experts and then carried out to families, which is ironic given that the concept of family-centred care emerged from a strong family advocacy movement” may also be apt in Indigenous settings. This finding may be related to use of a definition of family-centredcare developed for health service (rather than broader community) settings. However, the finding suggests that there is an important opportunity to develop a model of Indigenous family-centred care in the wider community context.

We found only three studies which considered the value of family-centred approaches in responding to the upstream social and economic determinants of Indigenous people's relatively poor health. The paucity of evidence in this area is of concern given the tendency identified by Popay et al. [51] for policies and programs to lifestyle drift; that is, to recognise the importance of the structural/political determinants of health inequalities but to respond with action largely on behavioural lifestyle foci.

Another key gap identified in the reporting of intervention strategies pertained to the role of fathers in family-centred care. Ball [52] cited mother-centrism in parenting practices and child welfare services as barriers to positive involvement of Indigenous fathers with their children’s health and wellbeing, yet none of the included studies explicitly considered the role of fathers. Further evaluation of the role of fathers in family-centred care interventions is needed through effective partnerships between primary healthcare services and research agencies to evaluate family-centred interventions as they roll out, thus minimising evaluation costs and optimising the use of locally available resources.

Only one study provided evidence of the costs of providing family-centred care to Indigenous families [27], and suggested that costs were offset by potential benefits. The paucity of economic evaluations was an identified gap in the scoping review. Another study of an intervention where senior Aboriginal women provided cultural support to pregnant women from remote Australian communities during labour, which was excluded from the review because it did not continue past one month post-partum, also found that the intervention was likely to be cost effective [53]. The finding suggests the potential for such interventions to be cost effective, but further such evaluations are needed.

A crucial issue in translating the results of this scoping review into policy or practice to inform interventions for improved Indigenous family health is that while the scoping study mapped the research and found 18 studies, these were generally of moderate to weak quality. This scoping review was conducted to produce a broad map of the evidence and to inform the scope and research objective of a Cochrane review protocol [3]. The Cochrane review will provide an independent and rigorous investigation, updated regularly to incorporate new research, of the best available evidence for the effects of family-centred interventions for children and their families. The Cochrane review will ensure that primary healthcare services can base their decisions about optimal interventions for the improvement of families’ health on current and reliable evidence.

## Conclusion

Family-centred interventions produced outcomes of improving Indigenous early childhood wellbeing, and the health of parents/ caregivers, as well as consumer satisfaction and improved access to mainstream services. The 18 studies evaluated or described the required conditions for implementing family-centred care to be the availability of competent and compassionate program deliverers, flexibility of access, continuity and integration of healthcare and culturally supportive care. Strategies were diverse and included supporting family behaviours and self-care, increasing maternal knowledge, strengthening links with the clinic, building the Indigenous workforce, promoting cultural or community connectedness and advocating for the social determinants of health. However, the evidence base for family-centred interventions by primary healthcare services is in an early stage of development, with few impact evaluation studies available. As well, there was little explanation in the available studies of how families engaged with and were positioned within family-centred interventions, whether or how interventions were able to impact the social determinants of families’ health, the role of fathers in family-centred care and the costs of providing family-centred care. This scoping review informs the development of a Cochrane review protocol, which will provide regular updates of the available evidence as it develops.

## Abbreviations

ISAC: Centre for Research Excellence for Improving Health Services for Aboriginal and Torres Strait Islander Children
PRISMA: Preferred Reporting Items for Systematic Reviews and Meta-Analyses

## References

[1] Anderson I, Robson B, Connolly M, Al-Yaman F, Bjertness E, King A (2016). Indigenous and tribal peoples’ health (The Lancet–Lowitja Institute Global Collaboration): a population study. Lancet.

[2] United Nations. State of the World’s Indigenous Peoples. New York: Indigenous Peoples’ Access to Health Services; 2016.

[3] McCalman J, Campbell SK, Chamberlain C, Strobel NA, Bainbridge RG, Wenitong M et al. Family- centred interventions for Indigenous early childhood well being by primary healthcare services (Protocol). Cochrane Database Syst Rev. 2016. 10.1002/14651858.CD012463.pub2 PMC9746601 36511823

[4] Colquhoun HL, Levac D, O'Brien KK, Straus S, Tricco AC, Perrier L (2014). Scoping reviews: time for clarity in definition, methods, and reporting. J Clin Epidemiol.

[5] Dijkers M (2015). What is a Scoping Review?. KT Update.

[6] Kuo DZ, Houtrow AJ, Arango P, Kuhlthau KA, Simmons JM, Neff JM (2012). Family-centered care: current applications and future directions in pediatric health care. Matern Child Health J.

[7] US Department of Human Services. Healthy People. US Department of Human Services. Washington, DC. 2016. https://www.healthypeople.gov/. 2016. Accessed 13 Feb 2017.

[8] Bamm EL, Rosenbaum P (2008). Family-centered theory: origins, development, barriers, and supports to implementation in rehabilitation medicine. Arch Phys Med Rehabil.

[9] Smylie J, Adomako P. Indigenous children’s health report. Centre for Research on Inner City Health. 2009.

[10] Libesman T. Child welfare approaches for Indigenous communities: International perspectives. Toronto: Keenan Research Centre, The Centre of Research on Inner City Health; 2004. p. 11-66. http://www.crich.ca. Accessed 12 Feb 2017.

[11] Silburn S, Zubrick S, De Maio J, Shepherd C, Griffin J, Mitrou F (2006). The Western Australian Aboriginal Child Health Survey: strengthening the capacity of Aboriginal children, families and communities.

[12] Nixon J. Family cohesion in families with an impaired child. Brisbane: University of Queensland; 1989.

[13] Griew R, Tilton E, Stewart J. Family centred primary health care: review of evidence and models funded by the Office for Aboriginal and Torres Strait Islander Health Department of Health and Ageing. Canberra: Robert Griew Consulting with JTAI Pty Ltd.; 2007.

[14] Institute of Medicine. Crossing the Quality Chasm: A new Health System for the 21st Century. Brisbane: The National Academies Press; 2001. 25057539

[15] Shields L, Zhou H, Pratt J, Taylor M, Hunter J, Pascoe E. Family‐centred care for hospitalised children aged 0–12 years. Washington: The Cochrane Library; 2012. p. 1-59. 10.1002/14651858.CD004811.pub3 PMC11531909 23076908

[16] Trivette CM, Dunst CJ, Allen S, Wall L (1993). Family-centeredness of the Children's Health Care journal. Child Health Care.

[17] Effective Public Health Practice Project. Quality assessment tool for quantitative studies. 2004. Accessed 2 Sept 2016.

[18] Critical Appraisal Skills Programme (2013). Critical Appraisal Skills Programme: making sense of evidence.

[19] Wolfswinkel JF, Furtmueller E, Wilderom CP (2013). Using grounded theory as a method for rigorously reviewing literature. Eur J Inf Syst.

[20] Strauss A, Corbin J. Basics of qualitative research: techniques and procedures fordeveloping grounded theory. Thousand Oaks: Sage Publications, Inc; 1998.

[21] Moher D, Liberati A, Tetzlaff J, Altman DG (2009). Preferred reporting items for systematic reviews and meta-analyses: the PRISMA statement. Ann Intern Med.

[22] Barlow A, Mullany B, Neault N, Compton S, Carter A, Hastings R (2013). Effect of a paraprofessional home-visiting intervention on American Indian teen mothers’ and infants’ behavioral risks: a randomized controlled trial. Am J Psychiatry.

[23] Barlow A, Mullany B, Neault N, Goklish N, Billy T, Hastings R (2015). Paraprofessional-delivered home-visiting intervention for American Indian teen mothers and children: 3-year outcomes from a randomized controlled trial. Am J Psychiatry.

[24] Barlow A, Varipatis-Baker E, Speakman K, Ginsburg G, Friberg I, Goklish N (2006). Home-visiting intervention to improve child care among American Indian adolescent mothers: a randomized trial. Arch Pediatr Adolesc Med.

[25] Mullany B, Barlow A, Neault N, Billy T, Jones T, Tortice I, et al. The Family Spirit Trial for American Indian teen mothers and their children: CBPR rationale, design, methods and baseline characteristics. Prev Sci. 2012;13(5):504–18. doi:10.1007/s11121-012-0277–2. 10.1007/s11121-012-0277-2 22932743

[26] Walkup JT, Barlow A, Mullany BC, Pan W, Goklish N, Hasting R (2009). Randomized controlled trial of a paraprofessional-delivered in-home intervention for young reservation-based American Indian mothers. J Am Acad Child Adolesc Psychiatry.

[27] Edmunds K, Searles A, Neville J, Ling R, McCalman J, Mein J. Apunipima Baby Basket Program: a retrospective cost study. BMC Pregnancy Childbirth. In press. 10.1186/s12884-016-1133-3 PMC5094003 27809875

[28] McCalman J, Edmunds K, Jongens C, Wargent R, Bainbridge R, Ling R (2014). Evaluating the Baby Basket program in north Queensland: as delivered by Apunipima Cape York Health Council, 2009 to 2013.

[29] McCalman J, Searles A, Bainbridge R, Ham R, Mein J, Neville J (2015). Empowering families by engaging and relating Murri way: a grounded theory study of the implementation of the Cape York Baby Basket program. BMC Pregnancy Childbirth.

[30] Turner K, Sanders M (2007). Family intervention in Indigenous communities: emergent issues in conducting outcome research. Australas Psychiatry.

[31] Turner KM, Richards M, Sanders MR (2007). Randomised clinical trial of a group parent education programme for Australian Indigenous families. J Paediatr Child Health.

[32] Abel S, Stockdale-Frost A, Rolls R, Tipene-Leach D (2015). The wahakura: a qualitative study of the flax bassinet as a sleep location for New Zealand Maori infants. N Z Med J.

[33] Atkinson R (2001). Antenatal care and perinatal health–how to do it better in an urban Indigenous community.

[34] Ball J (2005). Early childhood care and development programs as hook and hub for inter-sectoral service delivery in First Nations communities. J Aboriginal Health.

[35] Black AP, Vally H, Morris PS, Daniel M, Esterman AJ, Smith FE (2013). Health outcomes ofa subsidised fruit and vegetable program for Aboriginal children in northern New South Wales. Med J Aust.

[36] Blinkhorn F, Brown N, Freeman R, Humphris G, Martin A, Blinkhorn A (2012). A phase II clinical trial of a dental health education program delivered by aboriginal health workers to prevent early childhood caries. BMC Public Health.

[37] D'Espaignet ET, Measey ML, Carnegie MA, Mackerras D (2003). Monitoring the ‘Strong Women, Strong Babies, Strong Culture Program’: the first eight years. J Paediatr Child Health.

[38] Elliott E, Latimer J, Fitzpatrick J, Oscar J, Carter M (2012). There's hope in the valley. J Paediatr Child Health.

[39] Harvey-Berino J, Rourke J (2003). Obesity Prevention in Preschool Native-American Children: A Pilot Study Using Home Visiting. Obes Res.

[40] Homer CS, Foureur MJ, Allende T, Pekin F, Caplice S, Catling-Paull C (2012). ‘It’s more than just havinga baby’ women’s experiences of a maternity service for Australian Aboriginal and Torres Strait Islander families. Midwifery.

[41] Munns A (2010). Yanan Ngurra-ngu Walalja Halls Creek Community Families Programme. Neonatal. Paediatr Child Health Nurs.

[42] Poole N. Evaluation report of the Sheway Project for high-risk pregnant and parenting women. Vancouver: British Columbia Centre of Excellence for Women's Health; 2000.

[43] Applequist KL, Bailey DB (2000). Navajo caregivers’ perceptions of early interventionservices. J Early Interv.

[44] Di Lallo S (2014). Prenatal care through the eyes of Canadian Aboriginal women. Nurs Womens Health.

[45] Thomas S, Williams K, Ritchie J, Zwi K (2015). Improving paediatric outreach services for urban Aboriginal children through partnerships: views of community-based service providers. Child Care Health Dev.

[46] Arney F, Bowering K, Chong A, Healy V, Volkmer B (2010). Sustained nurse home visiting with families of Aboriginal children. Working with vulnerable families: A partnership approach.

[47] Easterbrook PJ, Gopalan R, Berlin J, Matthews DR (1991). Publication bias in clinical research. Lancet.

[48] Paul CL, Sanson-Fisher R, Stewart J, Anderson AE (2010). Being sorry is not enough: the sorry state of the evidence base for improving the health of indigenous populations. Am J Prev Med.

[49] Sanson-Fisher RW, Campbell EM, Perkins JJ, Blunden SV, Davis BB (2006). Indigenous health research:a critical review of outputs over time. Med J Aust.

[50] MacKean G, Thurston W, Scott C (2005). Bridging the divide between families and health professionals’ perspectives on family-centred care. Health Expect.

[51] Popay J, Whitehead M, Hunter DJ (2010). Injustice is killing people on a large scale—but whatis to be done about it?. J Public Health.

[52] Ball J (2009). Fathering in the shadows: Indigenous fathers and Canada's colonial legacies. Ann Am Acad Pol Soc Sc.

[53] Gao Y, Gold L, Josif C, Bar-Zeev S, Steenkamp M, Barclay L (2014). A cost-consequences analysis ofa Midwifery Group Practice for Aboriginal mothers and infants in the Top End of the Northern Territory, Australia. Midwifery.

[54] Edmunds K, Searles A, Neville J, Ling R, McCalman J, Mein J (2016). Apunipima Baby Basket Program: a retrospective cost study. BMC Pregnancy & Childbirth..

